# 4-Deoxy-l-*erythro*-5-hexoseulose Uronate (DEH) and DEH Reductase: Key Molecule and Enzyme for the Metabolism and Utilization of Alginate

**DOI:** 10.3390/molecules27020338

**Published:** 2022-01-06

**Authors:** Shigeyuki Kawai, Wataru Hashimoto

**Affiliations:** 1 Laboratory for Environmental Biotechnology, Research Institute for Bioresources and Biotechnology, Ishikawa Prefectural University, Nonoichi 921-8836, Ishikawa, Japan; 2 Laboratory of Basic and Applied Molecular Biotechnology, Division of Food Science and Biotechnology, Graduate School of Agriculture, Kyoto University, Uji 611-0011, Kyoto, Japan

**Keywords:** alginate, 4,5-unsaturated uronates, 4-deoxy-l-*erythro*-5-hexoseulose uronate (DEH), *Sphingomonas* sp. A1, DEH reductase, SDR superfamily, 2-keto-3-deoxy-d-gluconate (KDG), *Saccharomyces cerevisiae*, biorefinery, 2-furancarboxylic acid

## Abstract

4-Deoxy-l-*erythro*-5-hexoseulose uronate (DEH), DEH reductase, and alginate lyase have key roles in the metabolism of alginate, a promising carbon source in brown macroalgae for biorefinery. In contrast to the widely reviewed alginate lyase, DEH and DEH reductase have not been previously reviewed. Here, we summarize the current understanding of DEH and DEH reductase, with emphasis on (i) the non-enzymatic and enzymatic formation and structure of DEH and its reactivity to specific amino groups, (ii) the molecular identification, classification, function, and structure, as well as the structural determinants for coenzyme specificity of DEH reductase, and (iii) the significance of DEH for biorefinery. Improved understanding of this and related fields should lead to the practical utilization of alginate for biorefinery.

## 1. Introduction

**Figure 1:**
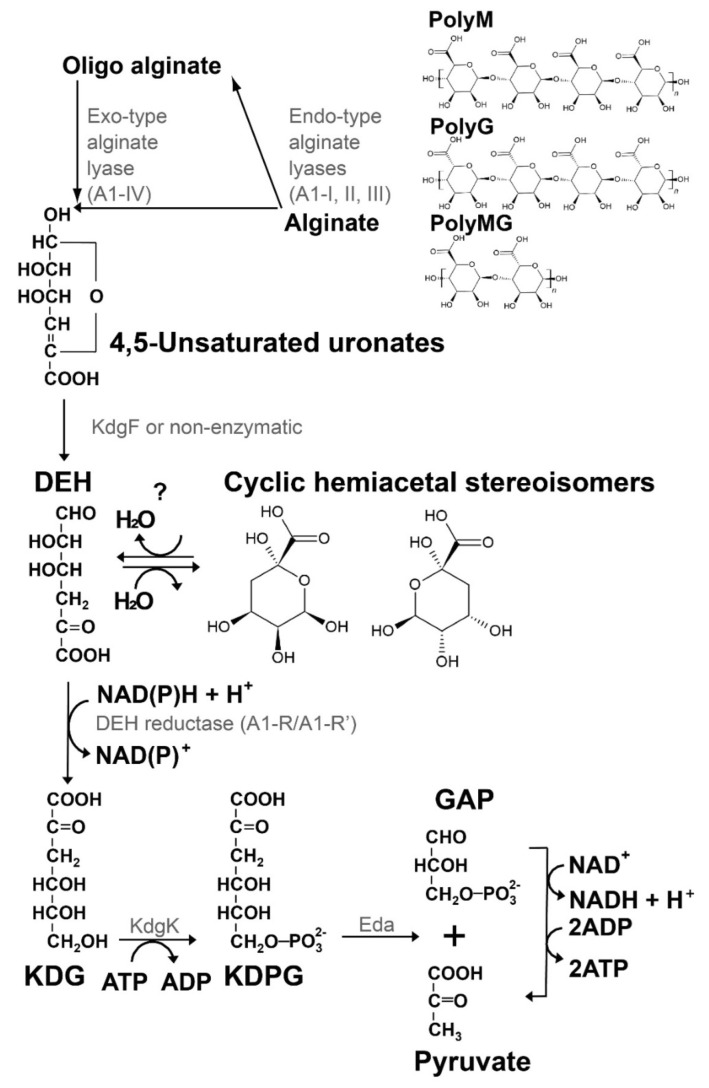
Pathway for alginate metabolism [1,8].

Brown macroalgae that contain up to 40% of alginate are a promising carbon source for biorefinery due to their advantages over terrestrial biomass including, e.g., the lack of requirements for agricultural fertilizer, pesticides, freshwater, and arable land [1]. Thus, understanding the metabolism of alginate is crucial for the utilization of brown macroalgae for biorefinery to produce fuels and chemicals. Alginate is a linear polyuronate consisting of D-mannuronic (M) and L-guluronic acid (G) residues [2]. The metabolic pathway of alginate is shown in Figure 1. Alginate is enzymatically degraded to 4,5-unsaturated uronates [4,5-unsaturated mannuronate (ΔManUA) and guluronate (ΔGulUA), which are structurally identical] by endo- and exo-type alginate lyases, and the monouronate is nonenzymatically or enzymatically converted to 4-deoxy-l-*erythro*-5-hexoseulose uronate (DEH) [3,4,5,6,7]. DEH is reduced to 2-keto-3-deoxy-d-gluconate (KDG) by DEH reductase, KDG is phosphorylated to 2-keto-3-deoxy-phosphogluconate (KDPG) by KDG kinase, and KDPG is cleaved into pyruvate and glyceraldehyde-3-phosphate (GAP) by KDPG aldolase [8,9,10]. In *Escherichia coli* that is unable to assimilate alginate, genes for KDG kinase (KdgK) and KDPG aldolase (Eda) are also found, and Eda is part of the Entner–Doudoroff pathway [11,12]. Thus, for the metabolism of alginate, the key molecule is DEH, and the key enzymes are alginate lyases and DEH reductase, which catalyze production and reduction of DEH, respectively. A number of reviews have described alginate lyases [13,14,15,16,17], but DEH and DEH reductase have not been previously reviewed. Thus, we focus on DEH and DEH reductase in this review.

## 2. Formation, Structure, and Reactivity of DEH

### 2.1. Non-Enzymatic Formation of DEH from 4,5-Unsaturated Uronate

In vitro, DEH is obtained by incubating sodium alginate with alginate lyase, using exo-type Atu3025 alone [18], exo-type Atu3025 plus endo-type A1-I [9], alginate lyase plus oligoalginate lyase [8], exo-type AlyFRB [19], or a crude extract from *Flavobacterium* sp. strain UMI-01 [20]. Alginate is enzymatically degraded to 4,5-unsaturated uronates [6]. The non-enzymatic conversion of 4,5-unsaturated uronates to DEH is thought to involve pyranose ring opening followed by enol-keto tautomerization [6], the whereas non-enzymatic formation of DEH can be detected by thin-layer chromatography (TLC) [19,20,21,22], liquid chromatography–mass spectroscopy (LC/MS) [19], and matrix-assisted laser desorption–ionization time-of-flight mass spectrometry (MALDI/TOF MS) [20,22]. DEH can be quantified by liquid chromatography–electrospray ionization–mass spectrometry (LC/ESI/MS) [23] or by the thiobarbituric acid method [7,24]. Shibata et al. [23] prepared DEH using AlyFRB and detected a peak in a selected ion monitoring (SIM) chromatogram for DEH at *m/z* 175, which corresponds to the molecular mass of the deprotonated ion of DEH in negative-mode LC/ESI/MS. Mori et al. [19] also analyzed AlyFRB products and detected a peak in the SIM chromatogram at *m/z* 176 in LC/MS. In other analyses of DEH, Inoue et al. [20] and Mochizuki et al. [20,22] detected the main peak at *m*/*z* 175 and a minor peak at *m*/*z* 176 using MALDI/TOF MS and found that both peaks decreased after reaction with DEH reductase.

### 2.2. Enzymatic Formation of DEH from 4,5-Unsaturated Uronate

DEH can be generated through an enzymatic reaction catalyzed by KdgF. Hobbs et al. [6] showed that purified recombinant *Ha*KdgF facilitates double bond depletion (disappearance of the absorbance at 230 nm (*A*_230_)) in a reaction mixture of dimannuronate [β-D-ManUA-(1–4)-D-ManUA], exo-type oligoalginate lyase Alg17c, and *Ha*KdgF. *Ha*KdgF is from the alginate-processing locus of a *Halomonas* sp. isolated from brown algae. In this system, dimannuronate without *A*_230_ was cleaved by Alg17c, yielding ΔManUA with a double bond (*A*_230_). Then, the disappearance of *A*_230_ suggests the facilitation of the formation of DEH in a ketonized form from ΔManUA, i.e., *Ha*KdgF catalyzes the formation of DEH from 4,5-unsaturated uronate.

Hobbs et al. [6] confirmed that purified recombinant *Ye*KdgF facilitates double bond depletion in a reaction mixture of digalacturonate [α-D-GalUA-(1–4)-D-GalUA], oligogalacturonate lyase from *Yersinia enterocolitica* (YeOgl), and *Ye*KdgF. In this system, digalacturonate without *A*_230_ was cleaved by YeOgl, yielding 4,5-unsaturated galacturonate (ΔGalUA) with a double bond (*A*_230_). ΔGalUA could be nonenzymatically converted to 5-keto-4-deoxyuronate (DKI) as 4,5-unsaturated uronates to DEH. Since DKI shows no *A*_230_, the disappearance of *A*_230_ again suggests facilitation of the formation of DKI in a ketonized form, i.e., *Ye*KdgF catalyzes the formation of DKI from ΔGalUA. Digalacturonate, ΔGalUA, and DKI are metabolites of pectin; thus, the pathways for the metabolism of pectin and alginate are parallel. *Y. enterocolitica* is a pectinolytic bacterium, and the gene for *Ye*KdgF is found on the pectinolytic locus of this bacterium. *Ye*KdgF and *Ha*KdgF share >50% amino acid sequence identity.

**Figure 2:**
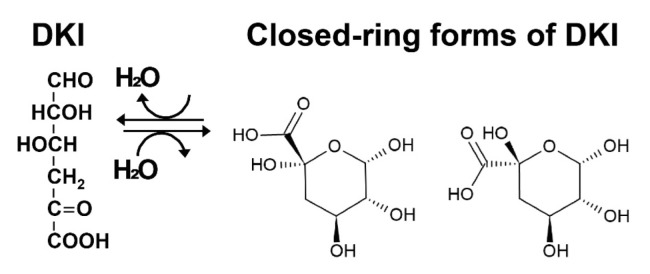
Closed-ring forms of DKI. 4-Deoxy-5-hydroxy-α-D-glucopyranuronate (**left**) and 4-deoxy-5-hydroxy-α-D-idopyranuronate (**right**) [6].

Hobbs et al. [6] showed that the reaction produced two pyranoses, as closed-ring forms of DKI, which were identified by one-dimensional selective total correlation NMR spectroscopy as 4-deoxy-5-hydroxy-α-D-glucopyranuronate and 4-deoxy-5-hydroxy-α-D- idopyranuronate (Figure 2). The same study also showed that a markerless KdgF deletion mutant of *E. coli* ATCC 25922 resulted in >40% reduction, but not complete loss, of the growth rate compared with wild-type ATCC 25922 (0.49·h^−1^ for wild type vs. 0.28·h^−1^ for the ΔkdgF strain) on endoacting polygalacturonate lyase-treated polygalacturonate (a main component of pectin). This supports the finding that DKI can be formed nonenzymatically in vivo and that KdgF facilitates the formation of DKI in vivo. Hobbs et al. [6] concluded that KdgF catalyzes the conversion of pectin- and alginate-derived 4,5-unsaturated monouronates to linear ketonized forms, a step in uronate metabolism that was previously thought to be spontaneous. In accordance with the significant role of KdgF in alginate metabolism, 21 of 26 alginate-utilizing marine bacterial strains isolated from Arctic and Antarctic marine environments have a gene for KdgF in alginate utilization gene clusters in genomic DNA [25].

### 2.3. DEH as a Substrate of DEH Reductase

DEH is a substrate of DEH reductase [20,21,22,26]. In the ESI/MS spectrum of the purified reaction product after incubation of DEH with DEH reductase A1-R [26], the main peak was at *m/z* 177.0 for the deprotonated ion [M-H]^−^ in negative ion mode. This shows that the molecular weight of the product corresponds to that of the reduced form (C_6_H_10_O_6_, MW = 178.05) derived from DEH (C_6_H_8_O_6_, MW = 176.03). ^1^H NMR and correlation spectroscopy (COSY) spectra also showed evidence that reduction of the aldehyde group at the carbon-1 position occurred in the A1-R product [26]. In vitro conversion of DEH into KDG by DEH reductase A1-R’ was shown by TLC analysis, in which an authentic KDG was used as a control [21]. Inoue et al. [20] and Mochizuki et al. [20,22] analyzed the conversion of DEH to KDG catalyzed by DEH reductases FlRed and HdRed with MALDI/TOF MS and detected a main peak at *m*/*z* 177, corresponding to deprotonated KDG, and a minor peak at *m/z* 178. Details of the A1-R and A1-R’ DEH reductases are described below [18,26].

### 2.4. Structure of DEH

The structure of DEH was initially elucidated by Preiss and Ashwell [7]. They partially purified “alginase from pseudomonad capable of utilizing alginic acid as sole carbon source” [7]. Sodium alginate was reacted with this partially purified enzyme for 90 min, and the reaction mixture was analyzed by paper chromatography. A compound with the fastest movement showed no ultra-violet absorption and increased in intensity in the reaction mixture incubated for 24 h. A large-scale reaction mixture was separated using a Dowex formate column, and the peaks were eluted by 0.15 N formic acid (peak I), followed by a gradient of ammonium formate (0 to 2.0 N) in water (peaks II and III). Peak I was the main compound (1.2 mmol recovered), peak II was a minor component (0.20 mol). Peak III was suggested to correspond to oligoalginates. Peaks I and II were indistinguishable on paper chromatography. The compounds giving rise to these peaks were identified as DEH using a chemical method and were enzymatically converted to KDG [7].

Enquist-Newman et al. [8] discovered a DEH transporter (Ac_DHT1) from the alginolytic eukaryote *Asteromyces cruciatus* and found unexpectedly that this transporter exhibited a high degree of similarity to the quinate transporter, despite the lack of structural similarity of DEH to quinate. To elucidate this finding, the structure of DEH in solution was analyzed using ^1^H-NMR, gradient COSY, and ^13^C-NMR, which demonstrated that DEH is mainly hydrated to form two cyclic hemiacetal stereoisomers, which do show structural similarity to quinate (Figure 1) [8], as in the case of DKI above [6] (Figure 2). Li et al. used ESI/MS, 1D ^1^H-NMR, ^13^C-NMR, and 2D ^1^H-^1^H COSY NMR to analyze the main products of the degradation of alginate (polyG block) with OalS6, an exo-type oligoalginate lyase from *Shewanella* sp. Kz7 [27]. The main products were two cyclic hemiacetal stereoisomers (2, 4, 5, 6-tetrahydroxytetrahydro-2H-pyran-2-carboxylic acid) formed by the hydration of DEH, as also found by Enquist-Newman et al. Li et al. confirmed that the main product was not DEH based on the lack of NMR signals for ketone and aldehyde [8]. However, as noted above, several groups detected DEH in a linear ketonized form with a molecular mass of 176 or 175 (deprotonated form) [19,20,22,23,26].

Based on all these findings, we propose that DEH can be reversibly hydrated to form two cyclic hemiacetal stereoisomers (Figure 1) that can serve as substrates for Ac_DHT1 and that DEH and the cyclic hemiacetal stereoisomers can coexist in equilibrium. The next question is the identity of the substrate of “DEH reductase”. As mentioned above, DEH reductase catalyzes the formation of KDG. If the cyclic hemiacetal stereoisomers are its substrates, DEH reductase has to catalyze both dehydration and reduction. Another possibility is that the hydration of DEH could occur reversibly (Figure 1), and thus, the substrate is still DEH. This may be in accord with the higher *K*_m_ of DEH compared to that of NADPH for DEH reductases A1-R (1930 vs. 9.55 µM) and A1-R’ (4790 vs. 15.5 µM) [18].

### 2.5. Reactivity of DEH with Specific Amino Groups

**Figure 3:**
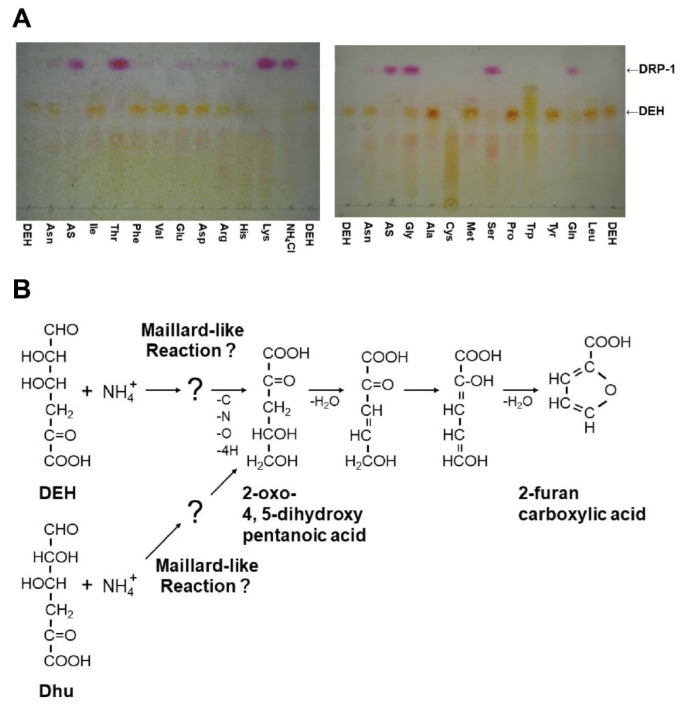
Reactions of DEH. (**A**) Reactivity of DEH with ammonium salts and amino acids. DEH (1% *w*/*v*, 56.8 mM) was incubated with 50 mM amino acids and ammonium salts at 30 °C for 24 h, with the exception of Tyr, Trp, and Asp, which were used at 1.0, 20, and 12.5 mM, respectively, because of their insolubility [21]. Figure modified from Figure 2A in ref. [21]. (**B**) Hypothetical pathway for the formation of 2-furancarboxylic acid from NH_4_^+^ and DEH or Dhu. Dhu is formed from a d-glucuronic acid residue via unsaturated glucuronic acid [28]. Figure modified from Figure 5 in ref. [21].

Nakata et al. [21] found that DEH reacts non-enzymatically with some amino groups. In incubations at 30 °C, DEH reacted with specific amino groups in Tris, ammonium salts [(NH_4_)_2_SO_4_ (AS) and NH_4_Cl], and certain amino acids (e.g., Gly, Ser, Gln, Thr, and Lys) and formed products including 2-furancarboxylic acid (Figure 3A). KDG, KDPG, and oligoalginate show no reactivity to ammonium salts. In the reaction with Tris at 30 °C, DEH disappeared very quickly after mixing with Tris-HCl; however, 2-furancarboxylic acid was generated only after 18 h of incubation. Regarding reactivity, Asn, Met, Glu, and Arg were almost inert and Ala, Pro, Leu, Ile, Phe, Val, and Asp, as well as sodium nitrate (NaNO_3_), were inert in the presence of DEH. Thus, some of these inert amino acids (Asn, Glu, Ala, Pro, Phe, and Asp) are suitable as a nitrogen source for a bioengineered yeast *Saccharomyces cerevisiae* with DEH-utilizing ability, as described below. In contrast, reactive amino acids (Ser, Gln, and Thr), as well as reactive ammonium salts (NH_4_^+^), are poor nitrogen sources. Commercial nutrient-rich mixtures such as tryptone and yeast extracts also react with DEH, but the formation of 2-furancarboxylic acids depends on the product. YP consisting of 1% (*w*/*v*) yeast extract and 2% (*w*/*v*) tryptone and even 10-fold diluted YP is a better nitrogen source than 5 mM Asn. Nakata et al. [21] proposed a hypothetical pathway for 2-furancarboxylic acid formation (Figure 3B). In this pathway, Nakata et al. hypothesize that DEH, rather than the cyclic hemiacetal stereoisomers, reacts with the amino group.

## 3. DEH Reductase

### 3.1. Discovery and Classification of DEH Reductase

As described above, Preiss and Ashwell first identified a NADPH-dependent reductase for alginate metabolism in *Pseudomonas* in 1962 [29]. This reductase catalyzes the reduction of DEH to KDG in the presence of a coenzyme, and the enzyme activity is significantly expressed in alginate-grown bacterial cells in comparison with glucose-grown cells. However, molecular cloning of the gene for the enzyme was not achieved for about 50 years until 2010, because DEH was considered to be an attractive compound after we realized the significance of the biofuel production from alginate.

**Figure 4:**
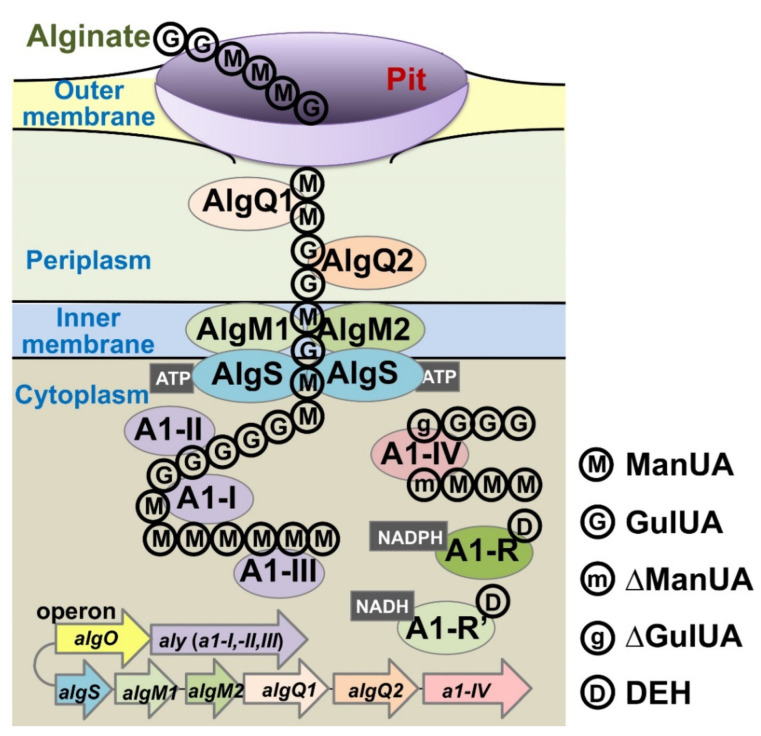
Schematic of import and degradation of alginate by strain A1. Extracellular alginate is concentrated in the cell surface pit and incorporated into the periplasm by an outer-membrane transporter. Periplasmic alginate-binding proteins (AlgQ1 and AlgQ2) deliver the polymer to an inner-membrane-bound ABC transporter (AlgM1–AlgM2/AlgS–AlgS). The ABC transporter incorporates alginate into the cytoplasm by energy generated through ATP hydrolysis. Alginate is degraded to constituent monosaccharides by endo-type (A1-I, A1-II, and A1-III) and exo-type (A1-IV) alginate lyases. The monosaccharides (unsaturated mannuronate and guluronate) are converted to DEH, which is reduced to KDG by DEH reductases (A1-R and A1-R’). Strain A1 operon genes for proteins and enzymes involved in import and degradation of alginate are expressed under the control of the transcriptional factor AlgO. Figure from ref. [36] with modifications.

The Gram-negative *Sphingomonas* sp. strain A1 is one of the most well-studied alginate-assimilating bacteria [30] (Figure 4). Strain A1 cells incorporate alginate polysaccharide through a cell surface pit and an ATP-binding cassette (ABC) transporter [31,32]. Periplasmic alginate-binding proteins (AlgQ1 and AlgQ2) deliver alginate from the pit to the ABC transporter, which has membrane-spanning domains (AlgM1 and AlgM2) and ATP-binding domains (two molecules of AlgS). The incorporated alginate is degraded to constituent monosaccharides through successive reactions of three endo-type alginate lyases (A1-I, A1-II, and A1-III) and an exo-type alginate lyase (A1-IV) [3,33] (Figure 1). Genes (IDs, *sph3807*-*3815*) encoding the proteins and enzymes for import and degradation of alginate are assembled in a cluster in the bacterial genome and inducibly transcribed in the presence of alginate under the control of the transcription factor AlgO, which is also encoded in the cluster [34]. The resultant monosaccharides (4,5-unsaturated uronates) from alginate are converted to DEH, as described above. Therefore, strain A1 is likely to be applicable to the production of biofuel and/or bioenergy [35].

To identify the gene coding for the DEH-converting enzyme, i.e., DEH reductase, the enzyme was purified from strain A1 cells to homogeneity for the molecular cloning of the gene. The purified enzyme (named A1-R) had a molecular mass of about 130 kDa (probably a homotetramer of 27 kDa subunits) and reduced DEH to KDG in the presence of NADPH [26]. A1-R acts with DEH, while other α-keto acids similar to DEH are inert with A1-R, and specifically prefers NADPH over NADH. These properties of A1-R are comparable with those obtained of the *Pseudomonas* enzyme [29]. The A1-R gene was identified as *sph3227* through peptide mass fingerprinting and is localized in the bacterial genome far from the gene cluster for alginate import/degradation. Based on a comprehensive DNA microarray analysis of gene expression in strain A1 cells, the A1-R gene was found to be constitutively expressed, although with slight inducible expression in the presence of alginate. A1-R consists of 258 amino acid residues and is classified as a member of the short-chain dehydrogenase/reductase (SDR) superfamily based on the primary structure. A coenzyme-binding motif Thr–Gly–X–X–X–Gly–X–Gly and catalytic triad (Tyr, Ser, Lys) conserved in SDR superfamily enzymes [37] are also found in A1-R.

Other DEH reductases have recently been identified in bacteria [20,38,39], abalone [22], and brown alga [40]. Bacterial DEH reductases belong to the SDR or β-hydroxy acid dehydrogenase (β-HAD) superfamily, whereas eukaryotic enzymes are members of the aldo–keto reductase (AKR) superfamily with a catalytic tetrad. In general, most SDR superfamily enzymes consist of four identical subunits (homotetramer) containing about 250 residues in each subunit with a catalytic triad or tetrad, whereas monomeric enzymes of ≥320 residues are common in the AKR superfamily [41]. Both SDR and AKR superfamily enzymes are physiologically important for the coenzyme-dependent metabolism of compounds such as aldehydes and ketones. The DEH reductase VsRed-1 of *Vibrio splendidus* belongs to the β-HAD superfamily containing a coenzyme-binding motif with a slight modification in the motif of SDR superfamily enzymes [39].

### 3.2. Structure of DEH Reductase

**Figure 5:**
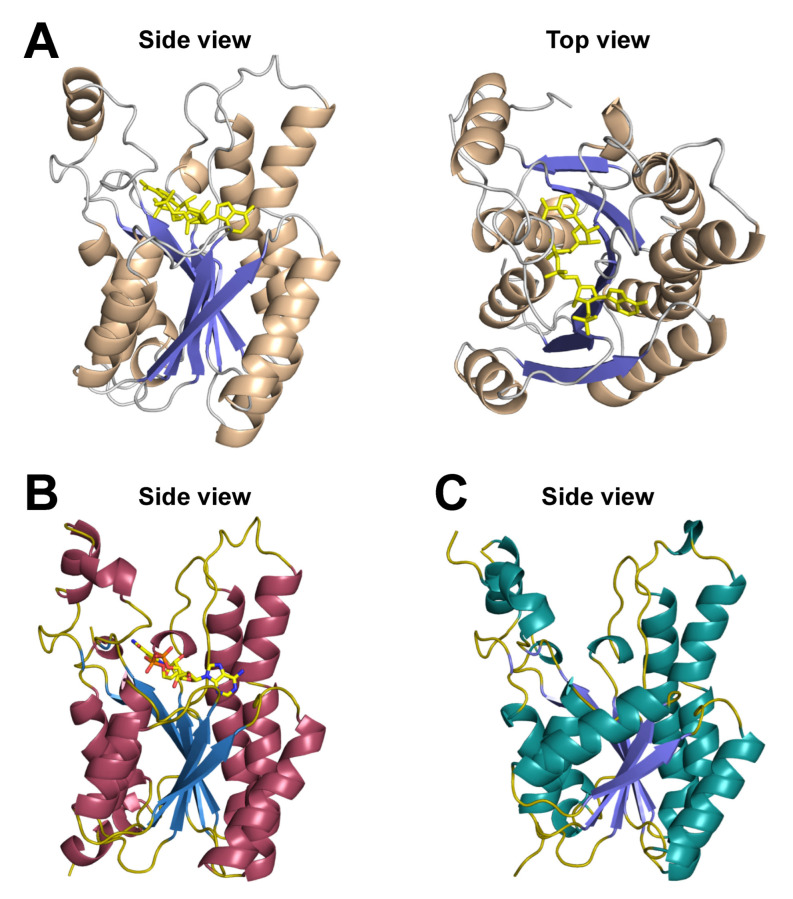
Crystal structure of SDR family enzymes. (**A**) NADP^+^-bound DEH reductase (A1-R) with a three-layered α/β/α structural fold [26]. α-helix, brown; β-strand, blue; loop and turn; gray; NADP^+^, yellow. (**B**) NAD^+^-bound KduD [43]. α-helix, purple; β-strand, blue; loop and turn; dark yellow; NADP^+^, yellow. (**C**) DhuD [28]. α-helix, cyan; β-strand, blue; loop and turn; dark yellow.

To determine the structural features of DEH reductase, the recombinant enzyme A1-R was overexpressed, purified, and crystallized. Crystal structures of A1-R and a NADP^+^-bound form (A1-R/NADP^+^) were determined at about 1.6 Å resolution by X-ray crystallography [26] (Figure 5A). The enzyme consists of 10 α-helices and 7 β-strands constituting a three-layered α/β/α structure as a basic scaffold. A Rossmann fold for coenzyme binding is included in A1-R, and NADP^+^ is bound to the Rossmann fold in A1-R/NADP^+^. As expected, A1-R has significant structural homology with some SDR superfamily enzymes, such as 3-oxoacyl reductase (PDB ID: 2UVD). Structure-based site-directed mutagenesis and kinetic parameters of the mutants demonstrated that Ser150, Tyr164, and Lys168 function as a catalytic triad for DEH reduction, and Ser16, Gly38, and Arg39 play important roles in the binding of NADP^+^, especially to the phosphate group.

DEH seems to be toxic to bacteria [42], suggesting that DEH reductase A1-R is essential and physiologically important for the immediate metabolism of DEH from alginate. Thus, to elucidate the physiological significance of A1-R, strain A1 mutant cells with low A1-R activity were constructed. An A1-R mutant (G38D) with Gly38 substituted with Asp exhibited significantly lower enzyme activity (4% of that of the wild-type enzyme) because Gly38 functions as a NADPH-binding residue. Unexpectedly, the strain A1 mutant cells producing the mutant A1-R (G38D) in place of native A1-R can grow on alginate as a sole carbon source and show enzyme activity for DEH reductase. These mutant cells were thought to produce another DEH reductase enzyme, and subsequently, distinct from A1-R, a DEH reductase designated as A1-R’ was found in strain A1 cells [18]. A1-R’ purified from strain A1 cells reduced DEH to KDG in the presence of NADH, rather than NADPH, indicating that A1-R’ is an NADH-dependent reductase.

### 3.3. Molecular Conversion of Coenzyme Specificity in DEH Reductase

**Figure 6:**
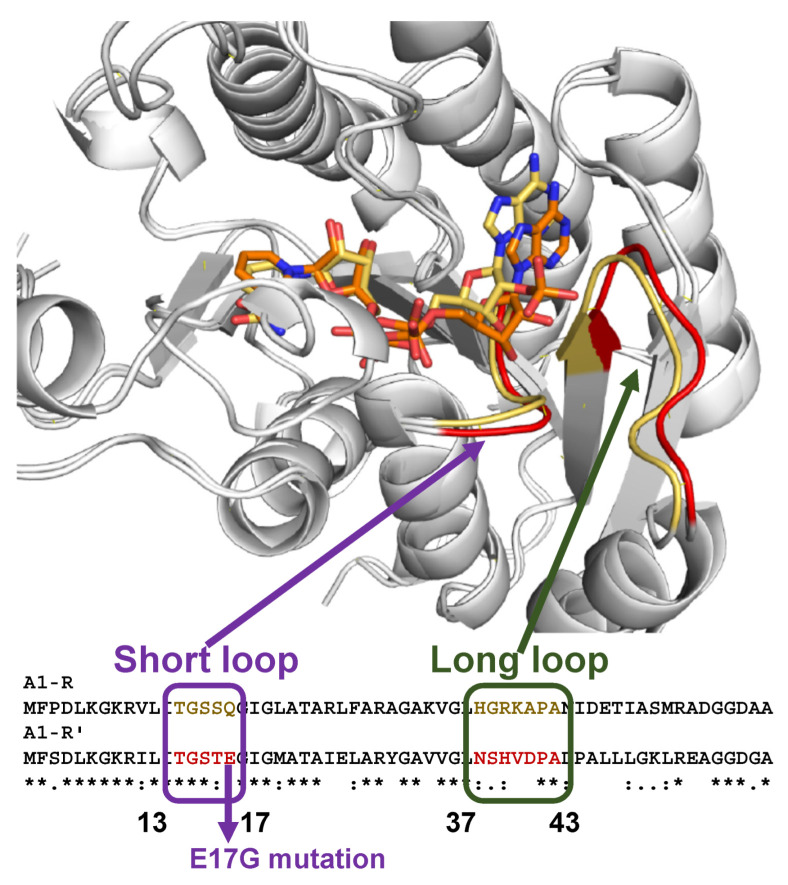
Structural determinants of coenzyme specificity. Two loops (short and long) are crucial for NAD^+^/NADP^+^ specificity. The two loops in A1-R and A1-R’ are colored yellow and red, respectively. Adaptively evolved yeast (*S. cerevisiae*) transformed with the A1-R’ gene produced the mutant A1-R’ with a Glu-to-Gly substitution at position 17 [9] as described below. Figure from ref. [18] with modifications.

After the determination of the N-terminal sequence of A1-R’, the gene coding for A1-R’ was found to be *sph1210* and to encode a polypeptide consisting of 258 amino acid residues. The A1-R’ gene is located in a different position, far from the genetic cluster for the import/degradation of alginate. There is significant sequence identity (64%) between A1-R and A1-R’, and both show similar enzyme properties, except for their coenzyme specificity (A1-R, NADPH; A1-R’, NADH). To examine the structural determinants for coenzyme specificity, the structures of ligand-free and NAD^+^-bound recombinant A1-R’ were determined by X-ray crystallography. A1-R’ also adopts a three-layered α/β/α structural fold, and A1-R and A1-R’ are almost entirely superimposable. There is, however, a structural difference in the coenzyme-binding sites of A1-R and A1-R’ for adenylate ribose at the 2′ position. The space in A1-R’ is clearly smaller in comparison with that in A1-R, and the narrow space in A1-R’ may prevent the accommodation of the phosphate group of NADPH. In addition, the molecular surface charge calculated at pH 7.0 shows that the corresponding site is positively charged in A1-R, but slightly negative in A1-R’, and this charge may be crucial for the binding or repulsion of the negatively charged NADPH phosphate. These differences in space and charge are caused by two loops, which are referred to as the short and the long loops (A1-R, short loop ^13^TGSSQ^17^, long loop ^37^HGRKAPA^43^; A1-R’, short loop ^13^TGSTE^17^, long loop ^37^NSHVDPA^43^), and the structural features in the two loops are probably important for coenzyme specificity (Figure 6).

The A1-R and A1-R’ mutants were constructed by exchange of the two loops, and the kinetic parameters of these mutants were determined to examine the function of the loops. The A1-R mutant containing two A1-R’-type loops showed NADH dependency, while the A1-R’ mutant with two A1-R-type loops became NADPH-dependent and exhibited higher enzyme activity than wild-type NADPH-dependent A1-R [18]. These results directly showed that the two loops are structural determinants of coenzyme specificity.

The short and long loops are conserved in SDR superfamily enzymes, and structural features caused by these loops have been found to be determinants of coenzyme specificity in two enzymes in this superfamily, KduD (Figure 5B) and DhuD (Figure 5C), for the metabolism of pectin and glycosaminoglycan, respectively [43]. *Pectobacterium carotovorum* KduD has an uncharged coenzyme-binding site that is intermediate in size between A1-R and A1-R’, and this enzyme uses both NADH and NADPH as a coenzyme. *Streptococcus pyogenes* DhuD contains a small and weakly negatively charged coenzyme-binding site, and this enzyme is specific for NADH. SDR superfamily enzymes are responsible for the metabolism of many biomolecules (e.g., sugars, fatty acids, steroids) in almost all organisms from bacteria to human, and the control of coenzyme balance is important for intrinsic activity in native cells and for the production of useful compounds related to biofuel and bioenergy in artificial recombinant cells. Therefore, the regulation of coenzyme specificity by the exchange of the two loops in SDR superfamily enzymes may contribute to the understanding of the physiological effects and to the establishment of industrial technology.

## 4. Significance of DEH for Biorefinery

### 4.1. Three Types of Hosts for the Utilization of Alginate in Biorefinery

Alginate has attracted attention as a carbon source for biorefinery [1,13,44]. Three types of microbial hosts have been proposed: (i) alginate-utilizing bacteria [35,45,46], (ii) alginate-non-utilizing bacteria [47], and (iii) alginate-non-utilizing yeast [8,9,10]. Among alginate-utilizing bacteria, *Sphingomonas* sp. A1 was used as a host for the production of ethanol [35], followed by the production of pyruvate [45], from alginate. The *Vibrio* sp. dhg was used as a host for the production of ethanol, 2,3-butanediol, and lycopene from alginate [46]. The alginate-non-utilizing bacterium *E. coli* was used as a host for production of ethanol from brown macroalgae, with the bacterium engineered to permit the assimilation of alginate [47]. In these bacterial systems, alginate or oligoalginate, but not DEH, enters the host and is metabolized. In contrast, in the alginate-non-utilizing yeast *S. cerevisiae*, DEH is significant as an intermediate of alginate metabolism and also for biorefinery itself, because DEH, but not alginate or oligoalginate, enters the host yeast cell [8,9,10].

### 4.2. Yeast as a Host for Biorefinery to Utilize Alginate

Three studies have used yeast *S. cerevisiae* as a host to produce ethanol from DEH or alginate [8,9,10]. In all three, four genes encoding DEH transporter (Ac_DHT1), DEH reductase (A1-R’ in our case), KDG kinase (KdgK), and aldolase (Eda) were introduced and functionally expressed in *S. cerevisiae*. Reactions catalyzed by A1-R’, KdgK, and Eda are shown in Figure 1. Enquist-Newman et al. first succeeded in creating DEH-utilizing *S. cerevisiae* [8]. Introduction and functional expression of the alginate transporter in *S. cerevisiae* is difficult because of the complexity of the bacterial alginate transport system. In an engineered *E. coli*, extracellular expression of a gene for endo-type alginate lyase and expression of genes for porin (KdgMN) enabled oligo-alginate to enter the periplasmic space, and genes for periplasmic alginate lyases (AlyABCD), a cell membrane symporter (ToaABC), and an intracellular exo-type oligoalginate lyase (OalABC) conferred the capacity to assimilate alginate [47].

Takagi et al. [10] introduced and functionally expressed exo-type alginate lyase on the cell surface of *S. cerevisiae*. Takagi et al. thus reported the production of ethanol from alginate and mannitol [10]. Enquist-Newman et al. produced ethanol from mixtures of DEH and mannitol [8], and our group evaluated the production of ethanol from DEH alone [9]. Enquist-Newman et al. and our group succeeded in the adaptive evolution of DEH-containing medium to improve the metabolism of DEH, and we also elucidated one of the mechanisms underlying this improvement. The mutation c.50A > G in the introduced DEH reductase (A1-R’) gene is one of the causes of improved metabolism; this mutation results in an E17G substitution in a short loop structure near the coenzyme-binding site of the reductase (Figure 6) and enhances reductase activity and aerobic growth in DEH-containing medium. We constructed DEH-utilizing *S. cerevisiae* in two strain backgrounds (BY4742 and D452-2) and obtained the adapted evolved strains in the two backgrounds. It should be noted that the same c.50A > G mutation was observed in both adapted strains in both backgrounds, emphasizing again the crucial role of the reductase reaction in the metabolism of DEH [9].

One of the critical points in the utilization of DEH as a carbon source for biorefinery is the reactivity of DEH with specific amino groups, as mentioned above [21]. Based on the amino acid composition of the brown macroalga *Saccharina latissima* [48] and its content of alginate [49], an estimated 0.014 mol of reactive amino acids (Gly, Ser, Gln, Thr, and Lys) and 0.18 mol of DEH could be generated from 100 g [dry weight (dw)] of *S. latissima* and could react, and thus, 7.8% (=100 × 0.014 mol/0.18 mol) DEH could be lost [21]. One solution to this problem is two step-fermentation, in which DEH is supplied only after the DEH-utilizing yeast metabolizes another carbon source, such as mannitol, to consume the reactive amino acids as a nitrogen source. Mannitol is also abundant (up to 23%) in brown macroalgae [1], and the ability to assimilate mannitol, in addition to DEH, has also been conferred to *S. cerevisiae* [8,9,10]. Because DEH showed no reactivity to sodium nitrate (NaNO_3_), the use of NaNO_3_ as a nitrogen source may be another solution. However, the nitrate utilization cluster [50] needs to be artificially expressed in *S. cerevisiae*, since the yeast is unable to assimilate nitrate as a nitrogen source [51].

## 5. Conclusions and Perspectives

In this review, we have summarized the current knowledge of DEH and DEH reductase and also raised several questions that require further study. Molecular identification and determination of the crystal structure of DEH reductase (A1-R/A1-R’) have paved the way to bioengineering microorganisms for alginate-based biorefinery and have provided insights into the classification and coenzyme specificity of the enzyme. The significance of DEH reductase has been proven from the adaptive evolution of a bioengineered yeast, whose increased activity improved the metabolism of DEH. Given the reactivity of DEH, it may be concluded that a bacterial system utilizing alginate, rather than DEH, is more suitable for biorefinery. However, a yeast system has several advantages for biorefinery, e.g., in the case of *S. cerevisiae*, robustness and high tolerance to ethanol and inhibitory compounds under process conditions, besides the considerable basic knowledge we have about this organism [52,53]. Thus, further studies of the practical utilization of DEH for biorefinery are warranted.

## Data Availability

Not applicable.
